# Association of delayed swallow screening with stroke-associated pneumonia and in-hospital outcomes in acute stroke patients in the neurocritical care unit

**DOI:** 10.3389/fmed.2026.1895263

**Published:** 2026-07-28

**Authors:** Bo Gao, Hongya Zhuo, Yingying Sun, Kun Zhang, Yujie Yang, Pan Wang, Runan Ren, Guolan Xu

**Affiliations:** The Neurocritical Care Unit, Zhengzhou People’s Hospital, Zhengzhou, Henan, China

**Keywords:** airway management, dysphagia screening, firth penalized regression, Neuro-ICU, restricted cubic spline, stroke-associated pneumonia

## Abstract

**Background:**

Stroke-associated pneumonia (SAP) is a frequent and devastating complication after acute stroke. Although early dysphagia screening is strongly recommended by guidelines, its implementation is often delayed in the Neurological Intensive Care Unit (Neuro-ICU). The precise continuous dose–response relationship between screening delay time and SAP risk has not been adequately quantified.

**Methods:**

We retrospectively enrolled 300 consecutive acute stroke patients admitted to the Neuro-ICU, excluding those requiring intubation within 24 h to avoid reverse causation. Hierarchical multivariable logistic regression, adjusting for consciousness level (Glasgow Coma Scale, GCS) and systemic inflammation (categorical Neutrophil-to-Lymphocyte Ratio, NLR) while excluding clinical mediators, evaluated the association between delayed screening (>24 h) and SAP. Restricted cubic spline (RCS) analysis modeled the non-linear dose–response relationship with 4 h as the reference point. Subgroup analyses used Firth’s penalized regression to address separation bias.

**Results:**

Overall SAP incidence was 48.3%. In the fully adjusted model, delayed screening remained an independent risk factor for SAP (adjusted OR = 3.69, 95% CI: 2.12–6.51, *p* < 0.001). The RCS curve showed a significant non-linear trajectory (*p* for non-linearity <0.05), with SAP odds remaining stable during the early buffer window but increasing steeply after the 24-h threshold. This effect was consistent across ischemic stroke (adjusted OR = 3.10, 95% CI: 1.59–6.10) and hemorrhagic stroke (adjusted OR = 4.69, 95% CI: 1.80–13.00) subgroups.

**Conclusion:**

Delayed dysphagia screening beyond 24 h was associated with a non-linear increase in the odds of SAP. The 24-h mark may represent a clinically relevant risk threshold, although residual confounding cannot be excluded.

## 1 Introduction

Acute ischemic and hemorrhagic strokes remain the leading causes of mortality and long-term disability worldwide (1, 2). Despite significant advances in acute reperfusion therapies—such as intravenous thrombolysis and endovascular thrombectomy—patients still face a high risk of debilitating complications after discharge (3). Among these, stroke-associated pneumonia (SAP) is one of the most common and devastating complications (4). SAP not only significantly prolongs the length of hospital stay and increases the economic burden on the healthcare system, but also serves as an independent risk factor for early post-stroke mortality and poor neurological recovery (5, 6). Therefore, identifying modifiable risk factors for SAP and implementing effective preventive strategies are crucial for optimizing clinical management and risk stratification in patients with acute stroke.

Post-stroke dysphagia is recognized as the core pathophysiological mechanism underlying the development of SAP, with an exceptionally high incidence during the acute phase of stroke (7, 8). Impaired swallowing function leads to silent or overt aspiration of oral secretions and gastric contents, which subsequently breaches the respiratory immune defense barrier and triggers pulmonary infection (9). Based on this well-established mechanism, the current guidelines for the early management of acute ischemic stroke from the American Heart Association/American Stroke Association (AHA/ASA) strongly recommend that all stroke patients undergo dysphagia screening using a validated tool before being given any oral intake of fluids, food, or medications (10). Substantial clinical evidence has demonstrated that early identification of dysphagia, coupled with prompt compensatory interventions such as nil-per-os (NPO) status, can significantly reduce the incidence of pneumonia and improve patient outcomes (11, 12).

However, in real-world clinical practice, particularly within the Neuro-Intensive Care Unit (Neuro-ICU), the implementation of early dysphagia screening frequently faces immense challenges (13). Patients admitted to the Neuro-ICU are typically critically ill, often presenting with severe disorders of consciousness, bulbar palsy, or the need for endotracheal intubation. These complex clinical scenarios frequently necessitate the postponement of the standard protocol, which dictates completing the screening within 24 h of admission (14). Previous studies based on large-scale stroke registries have clearly indicated that delays in dysphagia screening are significantly associated with an increased risk of SAP (15). Nevertheless, most of these studies treated the delay duration merely as a binary categorical variable, failing to systematically evaluate the continuous, non-linear “dose–response” relationship between the exact hours of delay and the subsequent risk of SAP. Furthermore, there is a lack of sufficient evidence regarding whether the prognostic value of delayed screening is modulated by different stroke pathological subtypes (i.e., ischemic vs. hemorrhagic).

Given these critical research gaps, this study aims to deeply investigate the prognostic value of delayed dysphagia screening for SAP occurrence and adverse clinical outcomes in patients with acute stroke in the Neuro-ICU. We hypothesized that delayed screening is associated with an increased risk of SAP, and that RCS modeling can describe the non-linear trajectory of this risk over time, thereby identifying a clinically relevant timeframe for intervention. Confirmation of this association would inform risk stratification and nursing protocols in critically ill stroke patients.

## 2 Materials and methods

### 2.1 Study design and population

A total of 324 patients were initially screened. After excluding 24 individuals who developed ventilator-associated pneumonia (VAP) secondary to prolonged intubation (>48 h), we enrolled a final consecutive sample of 300 acute stroke patients admitted to the Neuro-ICU between January 2024 and December 2025. This retrospective observational cohort study was designed, conducted, and reported in strict accordance with the Strengthening the Reporting of Observational Studies in Epidemiology (STROBE) guidelines to ensure methodological transparency.

The inclusion criteria were: (1) age ≥18 years; (2) diagnosis of acute ischemic or hemorrhagic stroke confirmed by computed tomography (CT) or magnetic resonance imaging (MRI); and (3) admission to the Neuro-ICU for intensive monitoring and care. Patients were excluded if they met any of the following criteria: (1) presence of active pulmonary infection or fever prior to admission; (2) history of severe pre-existing dysphagia or structural esophageal disease; (3) severe missing clinical data; (4) length of stay in the Neuro-ICU less than 24 h; or (5) requiring iatrogenic endotracheal intubation or invasive mechanical ventilation (IMV) within 24 h of admission, as these conditions physically precluded the possibility of standardized bedside swallowing screening and independently escalated the risk of ventilator-associated pneumonia (VAP).

During the screening phase, patients who developed pulmonary infections after receiving invasive mechanical ventilation (IMV) for more than 48 h were strictly classified as ventilator-associated pneumonia (VAP) and were completely excluded from the study sample prior to the final analysis. This target selection ensured that our final cohort of 300 individuals consisted exclusively of patients at risk for SAP, thereby preserving the epidemiological representativeness of non-VAP stroke complications. The study was conducted in accordance with the Declaration of Helsinki and approved by the Zhengzhou People’s Hospital Ethics Committee (Approval No.: 20260423). Written informed consent was obtained from all participants prior to their inclusion in the study, and all data were anonymized to protect participant privacy.

### 2.2 Definition of exposure and covariates

The primary exposure variable was the delay in dysphagia screening. Delayed screening was defined as a time interval of >24 h from Neuro-ICU admission to the completion of the first standardized bedside swallowing assessment (e.g., the water swallow test or the Gugging Swallowing Screen). Patients who underwent screening within 24 h constituted the non-delayed (control) group. Delay duration was also recorded as a continuous variable (in hours) for advanced dose–response modeling.

Baseline demographic and clinical covariates were extracted from the electronic medical records system. These included age, gender, stroke etiology, and the National Institutes of Health Stroke Scale (NIHSS) score at admission. Furthermore, the Glasgow Coma Scale (GCS) was included to reflect the baseline suppression of consciousness. To provide clinically interpretable effect sizes, the continuous Neutrophil-to-Lymphocyte Ratio (NLR) was transformed into a categorical variable (High vs. Low) based on a prespecified cutoff of 4.5.

### 2.3 Clinical outcomes

The primary study endpoint was the occurrence of stroke-associated pneumonia (SAP) during hospitalization. To avoid misclassification, patients who developed VAP (defined as pulmonary infection occurring after >48 h of invasive mechanical ventilation) were identified and excluded from the primary analysis, as detailed in the Study Population section.

SAP was rigorously diagnosed and stratified according to the specific modified Centers for Disease Control and Prevention (mCDC) criteria proposed by the Pneumonia in Stroke Consensus (PISCES) group. Based on this framework, cases were diagnosed combining systemic inflammatory criteria, respiratory symptom criteria, and new or progressive infiltrates/consolidations on serial chest radiography. The secondary endpoints included the Neuro-ICU length of stay (LOS) and the neurological functional outcome at discharge assessed via the modified Rankin Scale (mRS).

### 2.4 Statistical analysis

To guide covariate selection, we constructed a directed acyclic graph (DAG) based on presumed causal relationships (Supplementary Figure S1). Age, sex, admission NIHSS, GCS, and baseline NLR were identified as common causes of both delayed screening and SAP and were thus included as confounders. Nasogastric tube placement was considered a potential mediator on the causal pathway from delayed screening to SAP and was therefore excluded from the primary model to avoid over-adjustment bias. NLR was conceptualized primarily as a confounder reflecting pre-stroke immune status, and its possible mediating role was not formally tested.

All statistical analyses were performed using R software (version 4.x). Continuous variables were assessed for normality and presented as mean ± standard deviation (SD) or median with interquartile range [IQR], appropriately compared using Student’s t-test or the non-parametric Mann–Whitney *U* test. Categorical variables were expressed as frequencies and percentages, and between-group differences were evaluated using Pearson’s Chi-square test.

To evaluate the independent association between delayed screening (>24 h) and the risk of SAP, a robust multivariable logistic regression model was constructed, adjusting for predefined concept confounders including age, gender, admission NIHSS, GCS, and categorical NLR. Crucially, to prevent over-adjustment bias, nasogastric tube (NGT) placement was strictly excluded from the multivariable model as it acts as a clinical mediator following delayed screening.

To dynamically evaluate the non-linear dose–response relationship between delay duration and SAP risk, we utilized a Restricted Cubic Spline (RCS) model with 3 knots (placed at 15, 24, and 36 h). Crucially, the reference point (OR = 1.0) was pre-specified at 4 h to strictly align with the recommended operational window proposed by international acute stroke guidelines. Hierarchical models and Variance Inflation Factor (VIF) analyses were applied to rule out severe multicollinearity.

For subgroup analyses, to mitigate the risk of separation bias and overfitting due to small sample sizes (particularly in the hemorrhagic stroke subgroup, *n* = 93), Firth’s penalized likelihood logistic regression was employed. Results were reported as adjusted odds ratios with 95% confidence intervals.

## 3 Result

### 3.1 Baseline characteristics and clinical outcomes

A total of 324 patients were initially screened. After excluding 24 individuals who developed ventilator-associated pneumonia (VAP) secondary to prolonged intubation (>48 h), a final consecutive sample of 300 acute stroke patients admitted to the Neuro-ICU was enrolled. Among them, 146 (48.7%) patients experienced delayed dysphagia screening (>24 h), while 154 (51.3%) received screening within 24 h. As shown in Table 1, there were no significant differences between the delayed and non-delayed groups regarding age (*p* = 0.407) or gender distribution (*p* = 0.932). However, patients in the delayed screening group presented with significantly higher admission NIHSS scores (*p* < 0.001), lower Glasgow Coma Scale (GCS) scores (*p* < 0.001), a higher frequency of nasogastric tube (NGT) placement (*p* < 0.001), and a significantly larger proportion of patients with high systemic inflammation (NLR > = 4.5) (60.3% vs. 42.9%, *p* = 0.004). These differences reflect a substantially higher baseline burden of neurological severity and secondary systemic inflammation in the delayed cohort.

**Table 1:** Baseline characteristics and clinical outcomes of acute stroke patients stratified by dysphagia screening status.

Variables	Overall (*N* = 300)	Screened within 24 h (*N* = 154)	Screening Delay >24 h (*N* = 146)	*p* value
Demographics				
Age, years, mean ± SD	68.1 ± 9.7	68.5 ± 9.5	67.6 ± 10.0	0.407
Male sex, *n* (%)	140 (46.7%)	71 (46.1%)	69 (47.3%)	0.932
Clinical assessment				
Admission NIHSS, mean ± SD	15.0 ± 5.7	13.0 ± 5.3	17.2 ± 5.4	<0.001
GCS Score, mean ± SD	9.1 ± 2.6	9.9 ± 2.6	8.2 ± 2.6	<0.001
Stroke type, *n* (%)				
Ischemic stroke	207 (69.0%)	108 (70.1%)	99 (67.8%)	0.757
Hemorrhagic stroke	93 (31.0%)	46 (29.9%)	47 (32.2%)	
NGT Placement, *n* (%)	244 (81.3%)	111 (72.1%)	133 (91.1%)	<0.001
Systemic inflammation				
NLR (continuous), mean ± SD	4.5 ± 1.7	4.2 ± 1.6	4.9 ± 1.8	<0.001
NLR (categorical), *n* (%)				
Low NLR (<4.5)	146 (48.7%)	88 (57.1%)	58 (39.7%)	0.004
High NLR (≥4.5)	154 (51.3%)	66 (42.9%)	88 (60.3%)	
Outcomes				
SAP Development, *n* (%)	145 (48.3%)	43 (27.9%)	102 (69.9%)	<0.001
Neuro-ICU LOS, days, median (IQR)	14.0 (10.0–18.0)	11.0 (9.0–14.0)	16.0 (15.0–19.0)	<0.001
mRS at discharge, median (IQR)	4.0 (3.0–5.0)	3.0 (2.0–4.0)	5.0 (4.0–6.0)	<0.001

### 3.2 Association between delayed screening and SAP

**Figure 1:**
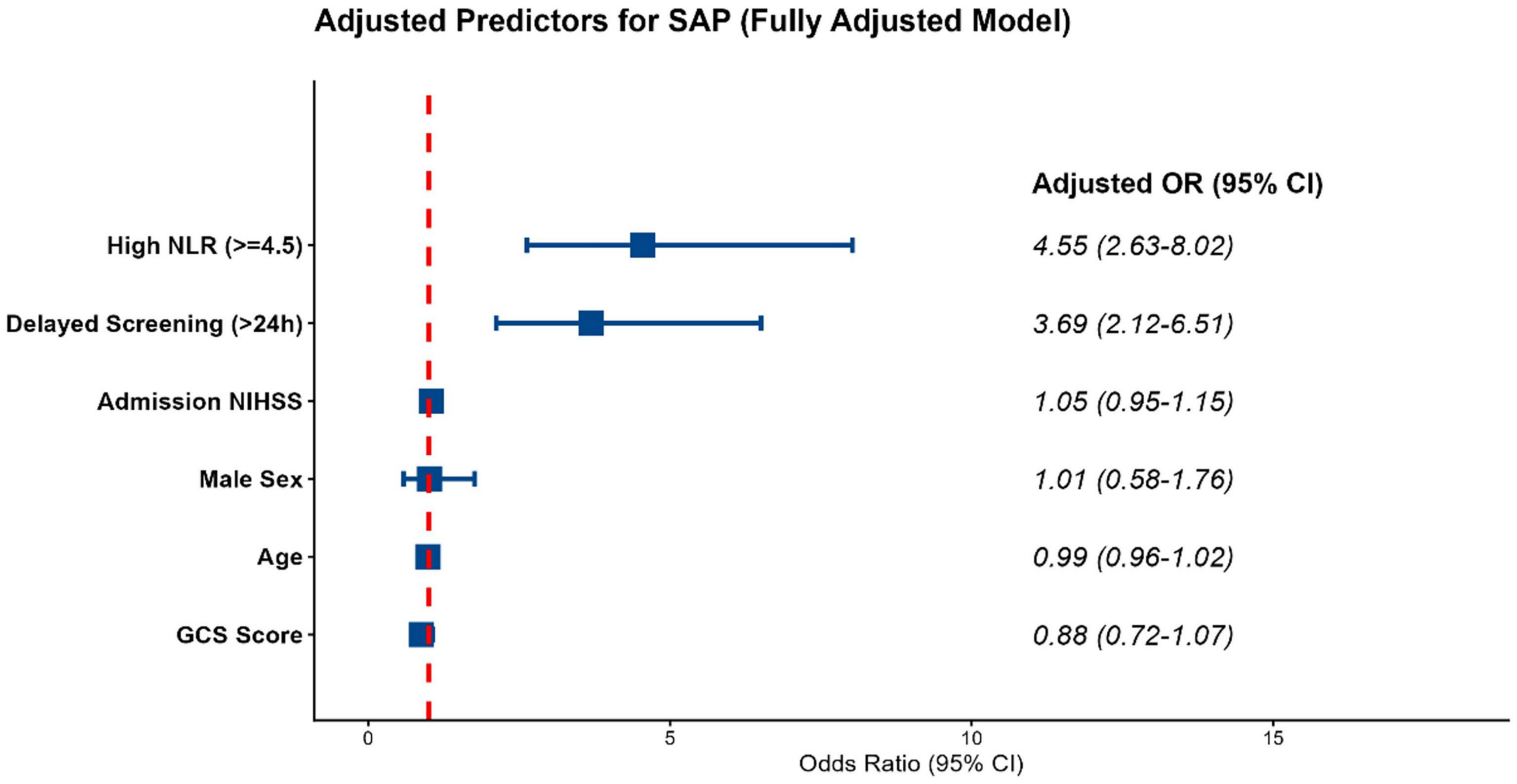
Forest plot of independent predictors for stroke-associated pneumonia.

The overall incidence of stroke-associated pneumonia (SAP) was 48.3% (145/300). Table 2 summarizes the hierarchical multivariable logistic regression models. In the basic model (Model 1), delayed screening (>24 h) and admission NIHSS demonstrated robust associations with an increased risk of SAP. Upon introducing categorical NLR into the full model (Model 2), the independent deleterious effect of delayed screening remained highly stable and significant (Adjusted OR = 3.69; 95% CI: 2.12–6.51; *p* < 0.001) (Figure 1). Variance Inflation Factor (VIF) analysis confirmed the absence of severe multicollinearity among the covariates (all VIFs <3.5). However, a notable attenuation in the significance of baseline neurological severity (NIHSS) was observed upon the incorporation of NLR. This attenuation is compatible with the hypothesis that NLR may partly lie on the causal pathway between stroke severity and SAP, although formal mediation analysis was not performed.

**Table 2:** Hierarchical multivariable logistic regression analysis of independent factors associated with SAP.

Variables	Model 1 (basic model)	*p-*value	Model 2 (full model)	*p*-value
	Adjusted OR (95% CI)		Adjusted OR (95% CI)	
Delayed screening (>24 h)	4.38 (2.60–7.48)	<0.001	3.69 (2.12–6.51)	<0.001
Age	0.99 (0.96–1.01)	0.309	0.99 (0.96–1.02)	0.417
Male sex	0.93 (0.55–1.56)	0.770	1.01 (0.58–1.76)	0.967
Admission NIHSS	1.08 (0.99–1.18)	0.087	1.05 (0.95–1.15)	0.338
GCS score	0.94 (0.79–1.13)	0.526	0.88 (0.72–1.07)	0.195
High NLR (≥4.5)	—	—	4.55 (2.63–8.02)	<0.001

Model 1 adjusted for baseline demographics and neurological severity. Model 2 additionally incorporated baseline systemic inflammation (categorical NLR). Variance Inflation Factor (VIF) analysis across the full model yielded values <5 for all covariates (max VIF = 3.35 for NIHSS), confirming the absence of severe multicollinearity.

The squares represent the adjusted Odds Ratios (ORs), and the horizontal lines represent the 95% Confidence Intervals (CIs). The precise adjusted OR and 95% CI for each variable are listed on the right. The red dashed vertical line indicates the null effect (OR = 1.0).

### 3.3 Non-linear dose–response relationship

**Figure 2:**
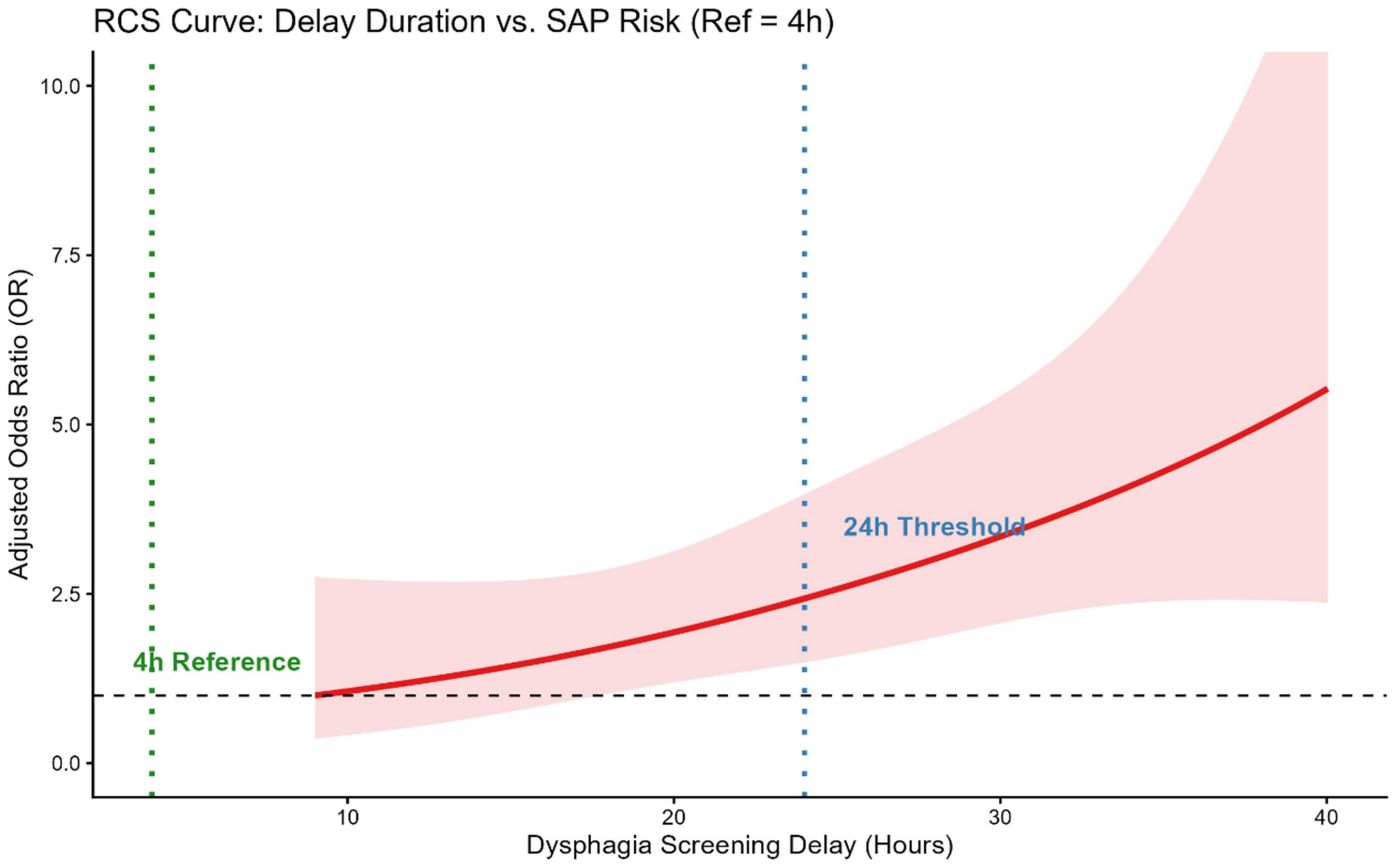
Restricted cubic spline (RCS) curve for the continuous association between screening delay duration and SAP risk.

To dynamically evaluate the non-linear dose–response relationship between delay duration and SAP risk, a Restricted Cubic Spline (RCS) model was employed, incorporating all the aforementioned critical covariates (Figure 2). The RCS curve revealed a significant non-linear association (*P* for non-linearity < 0.05). Using 4 h as the clinical reference point, the odds of SAP remained relatively stable at a plateau during the early physiological buffer window, but increased steeply after the 24-h threshold. This finding provides exploratory evidence for a clinically relevant timeframe for dysphagia assessment in critically ill stroke patients.

The solid red line represents the adjusted OR, and the shaded area represents the 95% CI. The horizontal dashed line marks the reference OR of 1.0, explicitly set at the 4-h clinical threshold. The vertical blue dotted lines highlight the 24-h point, after which the odds of SAP increase markedly.

### 3.4 Impact on hospital outcomes

The occurrence of SAP was associated with significantly poorer clinical outcomes (Supplementary Table S1). Length of Stay: Patients who developed SAP had a significantly longer median stay in the Neuro-ICU compared to those without SAP (16.0 days [IQR: 15.0–19.0] vs. 11.0 days [IQR: 9.0–14.0], *p* < 0.001). Neurological Disability: At discharge, patients in the SAP group exhibited significantly higher mRS scores (Median: 5.0 [IQR: 4.0–6.0]) compared to the non-SAP group (Median: 3.0 [IQR: 2.0–4.0], *p* < 0.001), indicating a profound burden of severe disability.

### 3.5 Subgroup and consistency analysis

**Figure 3:**
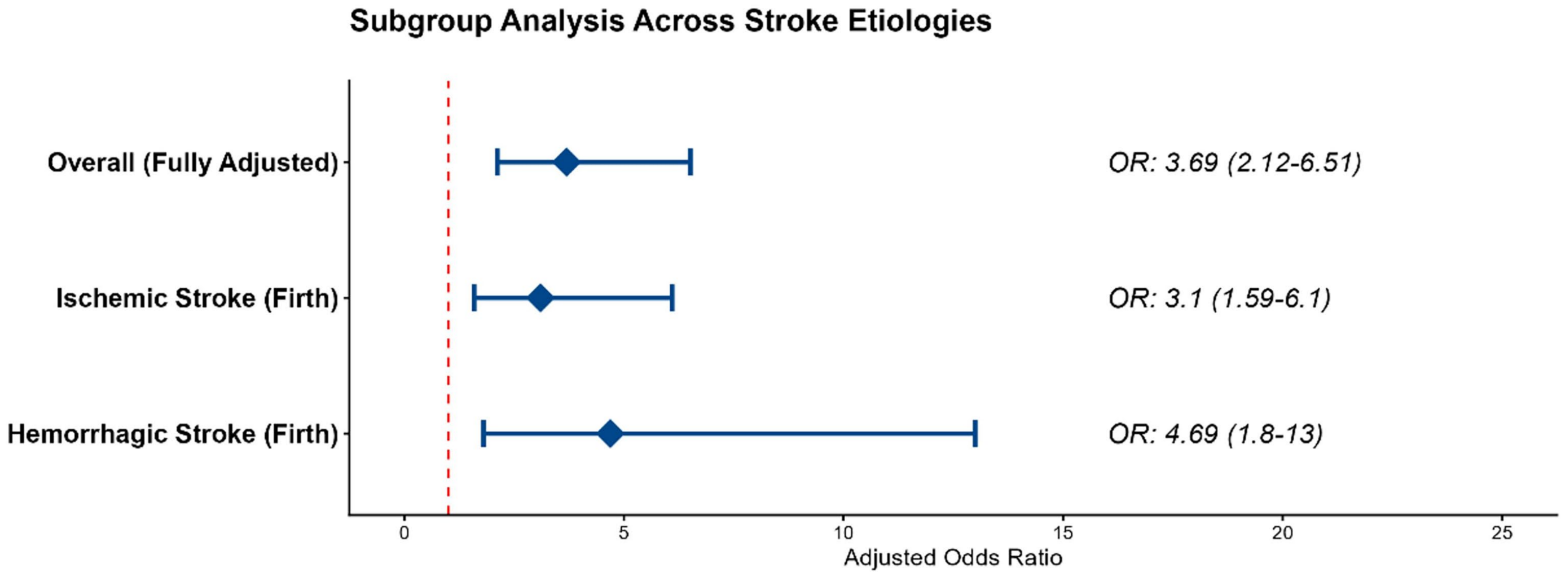
Subgroup forest plot showing consistency of the association across stroke etiologies.

To eliminate the risk of separation bias and overfitting caused by data sparsity in smaller subgroups (e.g., hemorrhagic stroke, *n* = 93), we introduced the highly advanced Firth’s penalized likelihood logistic regression for subgroup reconstruction (Table 3; Figure 3). Even after applying this most stringent statistical adjustment, the deleterious association between delayed screening and SAP remained highly consistent and significant in both the ischemic stroke (Adjusted OR = 3.10; 95% CI: 1.59–6.10, *p* < 0.001) and hemorrhagic stroke (Adjusted OR = 4.69; 95% CI: 1.80–13.00, *p* < 0.05) subgroups. Notably, the punitive effect of screening delay remained even more severe in the hemorrhagic stroke cohort, consistent with the hypothesis that aspiration-mediated pulmonary injury contributes substantially to SAP even in the context of primary intracranial hemorrhage.

**Table 3:** Subgroup analysis of the association between delayed screening and SAP.

Stroke etiology	Events/total (%)	Crude OR (95% CI)	Adjusted OR (95% CI)^**^	*p*-value
Overall (*N* = 300)	145/300 (48.3%)	5.94 (3.63–9.87)	3.69 (2.12–6.51)	<0.001
Delayed screening	102/146 (69.9%)	(Reference)		
Non-delayed screening	43/154 (27.9%)			
Ischemic stroke (*N* = 207)	104/207 (50.2%)	5.80 (3.17–10.87)	3.10 (1.59–6.10)	<0.001
Delayed screening	73/99 (73.7%)	(Reference)		
Non-delayed screening	31/108 (28.7%)			
Hemorrhagic Stroke (*N* = 93)^*^	41/93 (44.1%)	6.75 (2.56–19.46)	4.69 (1.80–13.00)	0.006
Delayed screening	29/47 (61.7%)	(Reference)		
Non-delayed screening	12/46 (26.1%)			

*The subgroup analysis for the Hemorrhagic Stroke cohort was conducted using Firth’s penalized likelihood logistic regression due to data sparsity in smaller sample sizes. **Adjusted for age, sex, admission NIHSS, GCS, and categorical NLR.

The diamonds represent the adjusted Odds Ratios (ORs), and the horizontal lines represent the 95% Confidence Intervals (CIs). The precise adjusted OR and 95% CI for each group are listed on the right. The red dashed vertical line indicates the null effect (OR = 1.0). To mitigate the risk of separation bias and overfitting in the smaller hemorrhagic stroke subgroup, the subgroup analyses were conducted using Firth’s penalized likelihood logistic regression.

### 3.6 Sensitivity analysis

To evaluate the stability of the association between delayed screening and SAP across key prognostic strata, we performed pre-specified sensitivity analyses stratified by NLR category and NIHSS severity (dichotomized at the median). In all subgroups, the direction and magnitude of the association remained robust (Supplementary Table S2). Firth’s penalized regression confirmed that delayed screening was consistently associated with an increased odds of SAP, with adjusted odds ratios ranging from 2.26 (95% CI: 0.98–5.22, *p* = 0.056) in the mild–moderate NIHSS group to 6.13 (95% CI: 2.84–13.97, *p* < 0.001) in the severe NIHSS group. The borderline significance in the mild–moderate subgroup is likely attributable to reduced sample size and lower event rate, and the overall pattern supports the robustness of the primary finding.

## 4 Discussion

In a high-risk cohort of acute stroke patients admitted to the Neuro-ICU, this study systematically validated the independent prognostic value of delayed dysphagia screening for SAP (14). Even after strictly excluding patients with early intubation to prevent reverse causation, omitting clinical mediators such as nasogastric tube placement, and adjusting for systemic consciousness suppression (GCS) and baseline immunological susceptibility (categorical NLR), a screening delay exceeding 24 h remained an independent predictor, likely reflecting both unmeasured severity and potentially modifiable aspiration risk (Adjusted OR = 3.69; 95% CI: 2.12–6.51) (13).

A key finding of this study is the 24-h point beyond which the odds of SAP increased more steeply revealed by the RCS model (16). Currently, international guidelines debate the optimal screening window, with the United Kingdom guidelines advocating a stringent 4-h standard (17). However, our RCS curve provides support for a Neuro-ICU-specific timeline: within the first 24 h (relative to the 4-h reference point), the risk of pneumonia maintains a stable baseline. This indicates that within this “early physiological buffer window,” neurocritical teams can safely prioritize hemodynamic stabilization and intracranial pressure management by maintaining a strict nil-per-os (NPO) status (9). However, once the 24-h screening vacuum is breached, the adjusted odds ratio increased steeply after 24 h.

Mechanistically, this observed non-linear increase may reflect the intersection of physical and immunological defense failures. According to the established AHA/ASA guidelines for the early management of acute stroke, early screening is no longer just about preventing aspiration; it is the prerequisite for initiating active neural remodeling therapies, such as Pharyngeal Electrical Stimulation (PES) (18). It has been hypothesized that prolonged NPO status without oral care may promote pathogenic bacterial colonization, and that acute brain injury can trigger a sympathetic storm and Stroke-Induced Immunodepression Syndrome (CIDS). The high prevalence of elevated NLR in the delayed group is consistent with this hypothesis, but the present study cannot confirm these mechanisms (19–21). The accumulation of micro-aspirations of highly virulent pathogens over 24 h may overwhelm alveolar macrophage function during a state of relative immunosuppression (22).

Furthermore, based on the robust Firth’s penalized regression model, our subgroup analysis revealed that the adjusted effect of delayed screening appeared more pronounced in the intracerebral hemorrhage (ICH) cohort (Adjusted OR = 4.69; 95% CI: 1.80–13.00) (23). However, the hemorrhagic stroke subgroup comprised only 93 patients with 41 SAP events, providing limited statistical power to detect interactions or to draw firm subtype-specific conclusions. These subgroup findings should be regarded as exploratory and hypothesis-generating. This highlights a dangerous “therapeutic fixation” in the Neuro-ICU, where critical airway protection is institutionally downgraded during the resuscitation of primary brain injury (24). These delays likely reflect systemic bottlenecks related to reliance on specialized Speech and Language Therapists (SALT). Our findings suggest that 24/7 nurse-led bedside screening protocols, such as those using the Gugging Swallowing Screen (GUSS), may help reduce iatrogenic risk exposure from interdisciplinary waiting times, though this requires prospective evaluation (11, 25).

This study has several limitations. First, the retrospective design inherently limits causal inference. Despite adjusting for GCS and NLR in both standard and Firth-penalized models, residual confounding by unmeasured factors—such as sedation depth, early goals of care, and oral care frequency—cannot be excluded. The 24-h threshold should therefore be viewed as a risk indicator rather than a definitively causal cutoff. Second, screening delay was modeled as a fixed baseline exposure. Patients who remained pneumonia-free longer had more opportunity to be classified as delayed, potentially introducing immortal time bias and overestimating the observed association. Third, this is a single-center Neuro-ICU study of 300 patients; the findings may not generalize to comprehensive stroke centers, community hospitals, or non-ICU stroke populations. Future multicenter studies with time-to-event methods and instrumental swallowing assessments such as FEES are needed to validate and refine the proposed threshold.

## Data Availability

The raw data supporting the conclusions of this article will be made available by the authors, without undue reservation.

## References

[1] Global, regional, and national burden of stroke and its risk factors, 1990-2019: a systematic analysis for the global burden of disease study 2019. Lancet Neurol. (2021) 20:795–820. doi: 10.1016/S1474-4422(21)00252-0, 34487721 PMC8443449

[2] Virani SS Alonso A Aparicio HJ Benjamin EJ Bittencourt MS Callaway CW . Heart disease and stroke Statistics-2021 update: a report from the American Heart Association. Circulation. (2021) 143:e254–743. doi: 10.1161/CIR.0000000000000950, 33501848 PMC13036842

[3] Campbell BCV Khatri P. Stroke. Lancet. (2020) 396:129–42. doi: 10.1016/S0140-6736(20)31179-X, 32653056

[4] Kishore AK Vail A Chamorro A Garau J Hopkins SJ Di Napoli M . How is pneumonia diagnosed in clinical stroke research? A systematic review and meta-analysis. Stroke. (2015) 46:1202–9. doi: 10.1161/STROKEAHA.114.007843, 25858238

[5] Finlayson O Kapral M Hall R Asllani E Selchen D Saposnik G. Risk factors, inpatient care, and outcomes of pneumonia after ischemic stroke. Neurology. (2011) 77:1338–45. doi: 10.1212/WNL.0b013e31823152b1, 21940613

[6] Hannawi Y Hannawi B Rao CP Suarez JI Bershad EM. Stroke-associated pneumonia: major advances and obstacles. Cerebrovasc Dis. (2013) 35:430–43. doi: 10.1159/000350199, 23735757

[7] Martino R Foley N Bhogal S Diamant N Speechley M Teasell R. Dysphagia after stroke: incidence, diagnosis, and pulmonary complications. Stroke. (2005) 36:2756–63. doi: 10.1161/01.STR.0000190056.76543.eb, 16269630

[8] Cohen DL Roffe C Beavan J Blackett B Fairfield CA Hamdy S . Post-stroke dysphagia: a review and design considerations for future trials. Int J Stroke. (2016) 11:399–411. doi: 10.1177/1747493016639057, 27006423

[9] Smith CJ Kishore AK Vail A Chamorro A Garau J Hopkins SJ . Diagnosis of stroke-associated pneumonia: recommendations from the pneumonia in stroke consensus group. Stroke. (2015) 46:2335–40. doi: 10.1161/STROKEAHA.115.009617, 26111886

[10] Powers WJ Rabinstein AA Ackerson T Adeoye OM Bambakidis NC Becker K . Guidelines for the early management of patients with acute ischemic stroke: 2019 update to the 2018 guidelines for the early management of acute ischemic stroke: a guideline for healthcare professionals from the American Heart Association/American Stroke Association. Stroke. (2019) 50:e344–418. doi: 10.1161/STR.0000000000000211, 31662037

[11] Palli C Fandler S Doppelhofer K Niederkorn K Enzinger C Vetta C . Early dysphagia screening by trained nurses reduces pneumonia rate in stroke patients: a clinical intervention study. Stroke. (2017) 48:2583–5. doi: 10.1161/STROKEAHA.117.018157, 28716980

[12] Titsworth WL Abram J Fullerton A Hester J Guin P Waters MF . Prospective quality initiative to maximize dysphagia screening reduces hospital-acquired pneumonia prevalence in patients with stroke. Stroke. (2013) 44:3154–60. doi: 10.1161/STROKEAHA.111.000204, 23963330

[13] Joundi RA Martino R Saposnik G Giannakeas V Fang J Kapral MK. Predictors and outcomes of dysphagia screening after acute ischemic stroke. Stroke. (2017) 48:900–6. doi: 10.1161/STROKEAHA.116.015332, 28275200

[14] Eltringham SA Kilner K Gee M Sage K Bray BD Pownall S . Impact of dysphagia assessment and management on risk of stroke-associated pneumonia: a systematic review. Cerebrovasc Dis. (2018) 46:99–107. doi: 10.1159/000492730, 30199856

[15] Bray BD Smith CJ Cloud GC Enderby P James M Paley L . The association between delays in screening for and assessing dysphagia after acute stroke, and the risk of stroke-associated pneumonia. J Neurol Neurosurg Psychiatry. (2017) 88:25–30. doi: 10.1136/jnnp-2016-313356, 27298147

[16] Harrell FE. Regression Modeling Strategies: with Applications to Linear Models, Logistic Regression, and Survival Analysis. Berlin: Springer (2001).

[17] GBD 2023 Disease and Injury and Risk Factor Collaborators. Burden of 375 diseases and injuries, risk-attributable burden of 88 risk factors, and healthy life expectancy in 204 countries and territories, including 660 subnational locations, 1990-2023: a systematic analysis for the global burden of disease study 2023. Lancet. (2025) 406:1873–922. doi: 10.1016/S0140-6736(25)01637-X, 41092926 PMC12535840

[18] Prabhakaran S Gonzalez NR Zachrison KS Adeoye O Alexandrov AW Ansari SA . 2026 guideline for the early management of patients with acute ischemic stroke: a guideline from the American Heart Association/American Stroke Association. Stroke. (2026) 50:e344–e418. doi: 10.1161/STR.0000000000000513, 41582814

[19] Tada A Miura H. Prevention of aspiration pneumonia (AP) with oral care. Arch Gerontol Geriatr. (2012) 55:16–21. doi: 10.1016/j.archger.2011.06.029, 21764148

[20] Nam KW Kim TJ Lee JS Kwon HM Lee YS Ko SB . High neutrophil-to-lymphocyte ratio predicts stroke-associated pneumonia. Stroke. (2018) 49:1886–92. doi: 10.1161/STROKEAHA.118.021228, 29967014

[21] Meisel C Schwab JM Prass K Meisel A Dirnagl U. Central nervous system injury-induced immune deficiency syndrome. Nat Rev Neurosci. (2005) 6:775–86. doi: 10.1038/nrn1765, 16163382

[22] Banda KJ Chu H Kang XL Liu D Pien LC Jen HJ . Prevalence of dysphagia and risk of pneumonia and mortality in acute stroke patients: a meta-analysis. BMC Geriatr. (2022) 22:420. doi: 10.1186/s12877-022-02960-5, 35562660 PMC9103417

[23] Wang Y Chen Y Chen R Xu Y Zheng H Xu J . Development and validation of a nomogram model for prediction of stroke-associated pneumonia associated with intracerebral hemorrhage. BMC Geriatr. (2023) 23:633. doi: 10.1186/s12877-023-04310-5, 37805464 PMC10559607

[24] Hess F Foerch C Keil F Seiler A Lapa S. Association of Lesion Pattern and Dysphagia in acute intracerebral hemorrhage. Stroke. (2021) 52:2921–9. doi: 10.1161/STROKEAHA.120.032615, 34000833

[25] Park KD Kim TH Lee SH. The Gugging swallowing screen in dysphagia screening for patients with stroke: a systematic review. Int J Nurs Stud. (2020) 107:103588. doi: 10.1016/j.ijnurstu.2020.103588, 32408200

