# Supplementary material for: Association of delayed swallow screening with stroke-associated pneumonia and in-hospital outcomes in acute stroke patients in the neurocritical care unit
Source: Front Med (Lausanne). 2026 Jul 28;13:1895263. doi: 10.3389/fmed.2026.1895263 (PMC13457306; doi:10.3389/fmed.2026.1895263)
Supplement: Supplementary file 1 [file Supplementary_file_1.docx]

**Supplementary Figure S1. Directed acyclic graph (DAG) of assumed causal relationships among variables.**

Abbreviations: Delay = Delayed screening (>24 h); SAP = Stroke-associated pneumonia; NIHSS = National Institutes of Health Stroke Scale; GCS = Glasgow Coma Scale; NLR = Neutrophil-to-lymphocyte ratio; NGT = Nasogastric tube placement.

Green node = exposure; Blue node = outcome; Grey nodes = confounders adjusted for in the primary model. NGT (dashed border) is a potential mediator and was excluded from the primary analysis. Red solid arrow = primary path of interest; Grey dashed arrows = mediation path.

**Supplementary Table S1. Comparison of hospital outcomes between patients with and without SAP.**

| Outcomes | Non-SAP (n = 155) | SAP (n = 145) | *P* value* |
| --- | --- | --- | --- |
| NICU LOS (days), Median [IQR] | 11.00 [9.00, 14.00] | 16.00 [15.00, 19.00] | < 0.001 |
| mRS at discharge, Median [IQR] | 3.00 [2.00, 4.00] | 5.00 [4.00, 6.00] | < 0.001 |

Note: Data are presented as median [interquartile range]. Abbreviations: NICU, Neurological intensive care unit; LOS, Length of stay; mRS, Modified Rankin Scale. P values were calculated using the Mann-Whitney U test.

**Supplementary Table S2. Sensitivity Analysis of the Association Between Delayed Screening and SAP Stratified by NLR and Stroke Severity**

| Subgroup | N | SAP Events | Adjusted OR (95% CI) | *P* value |
| --- | --- | --- | --- | --- |
| Stratified by NLR |  |  |  |  |
| High NLR (≥4.5) | 154 | 104 | 3.48 (1.66, 7.42) | < 0.001 |
| Low NLR (<4.5) | 146 | 41 | 3.58 (1.57, 8.35) | 0.002 |
| Stratified by NIHSS |  |  |  |  |
| Severe (NIHSS ≥15) | 161 | 101 | 6.13 (2.84, 13.97) | < 0.001 |
| Mild-Moderate (NIHSS <15) | 139 | 44 | 2.26 (0.98, 5.22) | 0.056 |
| All models are adjusted for age, sex, GCS, and either NLR or NIHSS as appropriate, using Firth's penalized likelihood logistic regression. | | | | |
